# NMR assignment of non-modified tRNA^Ile^ from *Escherichia coli*

**DOI:** 10.1007/s12104-022-10075-6

**Published:** 2022-03-11

**Authors:** Vanessa de Jesus, Thomas Biedenbänder, Jennifer Vögele, Jens Wöhnert, Boris Fürtig

**Affiliations:** 1 Institute for Organic Chemistry and Chemical Biology, Center for Biomolecular Magnetic Resonance (BMRZ), Johann Wolfgang Goethe-Universität, 60438 Frankfurt, Germany; 2 Institute of Chemistry and Department Life, Light & Matter, University of Rostock, 18059 Rostock, Germany; 3 Institute for Molecular Biosciences and Center for Biomolecular Magnetic Resonance (BMRZ), Johann Wolfgang Goethe-Universität, 60438 Frankfurt, Germany

**Keywords:** tRNA, RNA, NMR, Assignment, Modification, Dynamics

## Abstract

tRNAs are L-shaped RNA molecules of ~ 80 nucleotides that are responsible for decoding the mRNA and for the incorporation of the correct amino acid into the growing peptidyl-chain at the ribosome. They occur in all kingdoms of life and both their functions, and their structure are highly conserved. The L-shaped tertiary structure is based on a cloverleaf-like secondary structure that consists of four base paired stems connected by three to four loops. The anticodon base triplet, which is complementary to the sequence of the mRNA, resides in the anticodon loop whereas the amino acid is attached to the sequence CCA at the 3′-terminus of the molecule. tRNAs exhibit very stable secondary and tertiary structures and contain up to 10% modified nucleotides. However, their structure and function can also be maintained in the absence of nucleotide modifications. Here, we present the assignments of nucleobase resonances of the non-modified 77 nt tRNA^Ile^ from the gram-negative bacterium *Escherichia coli*. We obtained assignments for all imino resonances visible in the spectra of the tRNA as well as for additional exchangeable and non-exchangeable protons and for heteronuclei of the nucleobases. Based on these assignments we could determine the chemical shift differences between modified and non-modified tRNA^Ile^ as a first step towards the analysis of the effect of nucleotide modifications on tRNA’s structure and dynamics.

## Biological context

tRNA molecules play a central role in translation by decoding the information on the mRNA into a sequence of amino acids. During their lifetime, tRNAs encounter a variety of interaction partners: mRNAs, ribosomes, elongation factors, aminoacyl-tRNA synthetases and tRNA modification enzymes (Berg and Brandl 2021).

In all domains of life, tRNAs exhibit the same general secondary and tertiary structure. The secondary structure of tRNA is divided into five parts: the acceptor stem (Acc. stem), the dihydrouridine arm (D-arm), the anticodon-stem-loop (ACSL), the variable loop and the TΨC-arm. These elements form the well-known cloverleaf secondary structure. tRNA molecules contain a CCA-tail at the 3′-end, where the amino acid is covalently attached to the 2′- or 3′-OH group. They are charged with the corresponding amino acid matching the anticodon triplet by enzymes called aminoacyl-tRNA synthetases (aaRS), yielding aminoacyl-tRNA. The recognition sites for the aaRS are often nucleotides in the acceptor stem and the anticodon loop. In three dimensional space, the tRNA molecules adopt an L-shaped tertiary structure (Kim et al. 1973). In this highly folded structure the anticodon stem and the D-arm, as well as the acceptor stem and the TΨC-arm stack coaxially. These two stacks are positioned in the L-shape through long range interactions between the D- and TΨC-loops.

tRNAs constitute the class of RNAs with the highest modification rate and more than three quarters of all known types of modified nucleotides are found within their sequences (Boccaletto et al. 2018). The extensive post-transcriptional modification modulates their overall stability and translation efficiency (Lorenz et al. 2017). However, several studies have shown that the global structure can be also achieved in unmodified tRNA with Mg^2+^ concentration at and above 5 mM (Schauss et al. 2021). Therefore, it is very interesting to investigate the structural and dynamic changes that are induced by the modified nucleotides. In this respect, the structural description of modified and non-modified tRNAs is needed. Here, we present the assignments of nucleobase resonances of the non-modified 77 nt tRNA^Ile^ from *E. coli*.

The mature tRNA^Ile^ harbors in total nine modifications (Niimi et al. 1993): Dihydrouridines (D17, D20 and D20a), N^6^-threonylcarbamoyladenosine at position 37 (t^6^A37), 7-methylguanosine at position 46 (m^7^G46), 3-(3-amino-3-carboxypropyl)uridine at position 47 (acp^3^U47), ribothymidine (T54) and pseudouridines (Ψ55 and Ψ65). The comparison of chemical shifts of native and non-modified tRNA will provide the first indications of structural and dynamic changes that are induced through the modified nucleotides.

## Methods and experiments

### Sample preparation

The DNA template of 77 nt tRNA^Ile^ from *Escherichia coli* is flanked by two restriction sites and a hammerhead ribozyme:

GAATT (EcoRI)—TAATACGACTCACTATAG (T7 promotor)—GGACAAGCCTCTGATGAGTCCGTGAGGACGAAAGACCGTCTTCGGACGGTCTC (hammerhead ribozyme)—AGGCTTGTAGCTCAGGTGGTTAGAGCGCACCCCTGATAAGGGTGAGGTCGGTGGTTCAAGTCCACTCAGGCCTACCA (tRNA^Ile^)—TATG (NdeI).

Plasmid DNA and primer were purchased from Eurofins Genomics (Ebersberg, Germany). DNA was amplified by polymerase chain reaction (PCR) using T7 forward primer (5′-TAATACGACTCACTATAGG-3′) and tRNA^Ile^ reverse primer (5′-TGGTGCCCGGACTCG-3′).

^13^C-^15^N-labeled 77 nt tRNA^Ile^ was synthesized by in vitro transcription with T7 RNA polymerase from the PCR product as described in literature (Fürtig et al. 2003; Guilleres et al. 2005). The ^13^C-^15^N-labeled (rATP, rCTP, rGTP, rUTP) nucleotides for transcription were purchased from Silantes (Munich, Germany). tRNA^Ile^ was purified by preparative polyacrylamide gel electrophoresis according to standard protocols (Bains et al. 2019). The tRNA^Ile^ was folded in water by heat denaturation for 5 min at 95 °C and immediately diluted tenfold with ice-cold water. Afterwards, tRNA^Ile^ was buffer-exchanged into NMR buffer (25 mM potassium phosphate, 200 mM KCl, 5 mM MgCl_2_, pH 6.2).

### NMR spectroscopy

All experiments were performed in NMR buffer (25 mM potassium phosphate, 200 mM KCl, 5 mM MgCl_2_, pH 6.2) and 7% D_2_O with a sample concentration of 850 µM. All spectra were referenced to DSS (4,4-dimethyl-4-silapentane-1-sulfonic acid). Nitrogen-15 and Carbon-13 chemical shifts were indirectly referenced using the ratio of the gyromagnetic ratios of proton to ^15^N (0.101329118) and ^13^C (0.251449530), respectively (Wishart et al. 1995). All experiments were performed at 25 °C if not stated otherwise.

NMR experiments were performed on 600 MHz, 700 MHz, 800 MHz, 900 MHz or 950 MHz NMR spectrometers equipped with 5 mm, z-axis gradient: ^1^H {^13^C, ^15^N} TCI cryogenic probes (600 MHz, 800 MHz, 900 MHz, 950 MHz), a ^1^H {^13^C, ^15^N, ^31^P}-QCI cryogenic probe (700 MHz) or ^13^C, ^15^N {^1^H}-TXO cryogenic probe (800 MHz).

NMR experiments were analyzed using Bruker Biospin software TopSpin 3.5 and assignments were performed using the software NMRFAM-SPARKY (Lee et al. 2015).

To elucidate the base-pairing interactions and assign the resonances stemming from the imino and amino groups of the nucleotides as well as other ^1^H, ^15^N and ^13^C resonance from the nucleobases we conducted the following suite of NMR experiments (Table 1). To detect the homo-nuclear NOEs between imino protons and all other protons we performed a ^1^H, ^1^H-NOESY with jump-return water suppression (Stonehouse et al. 1994). The experiment was recorded on a 700 MHz NMR spectrometer at 25 °C with 130 ms and 170 ms mixing times and at 10 °C with 130 ms mixing time (Hwang and Shaka 1995; Sklenar 1995).

**Table 1:** List of NMR experiments with experimental parameters

	Field (MHZ)	Interscan delay (s)	No scans	Carrier frequencies	Spectral width	Acquisition time	Points
^1^H, ^1^H-NOESY	700	1.5	158	4.7 ppm (^1^H, t_2_) 7.5 ppm (^1^H, t_1_)	25 ppm (^1^H), 17 ppm (^1^H)	99 ms (^1^H), 21 ms (^1^H)	3494 (^1^H), 512 (^1^H)
^1^H,^15^ N-BEST-TROSY	700	0.2	16	4.7 ppm (^1^H), 153.5 ppm (^15^N)	25 ppm (^1^H), 30 ppm (^15^N)	80 ms (^1^H), 53 ms (^15^N)	2804 (^1^H), 228 (^15^N)
^1^H,^15^ N-CPMG-NOESY	600	1.5	258	4.7 ppm (^1^H), 116.0 ppm (^15^N)	25 ppm (^1^H), 100 ppm (^15^N)	99 ms (^1^H), 42 ms (^15^N)	2998 (^1^H), 512 (^15^N)
2D-^1^H,^15^ N-BEST-TROSY-HNN-COSY	600	0.3	400	4.7 ppm (^1^H), 185.0 ppm (^15^N)	21 ppm (^1^H), 120 ppm (^15^N)	63 ms (^1^H), 24 ms (^15^N)	1584 (^1^H), 360 (^15^N)
^1^H,^15^ N-sfHMQC	800	1.0	304	4.7 ppm (^1^H), 145.0 ppm (^15^N)	10 ppm (^1^H), 80 ppm (^15^N)	63 ms (^1^H), 18 ms (^15^N)	1024 (^1^H), 236 (^15^N)
^13^C-^15^ N-HSQC	800	2.0	512	4.7 ppm (^1^H), 155.0 ppm (^13^C), 85.75 ppm (^15^N)	70 ppm (^13^C), 31.5 ppm (^15^N)	36 ms (^13^C), 26 ms (^15^N)	1024 (^13^C), 136 (^15^N)
^1^H,^15^ N-HSQC	800	0.8	368	4.7 ppm (^1^H), 86.5 ppm (^13^C), 101 ppm (^15^N)	16 ppm (^1^H), 38 ppm (^15^N)	40 ms (^1^H), 41.5 ms (^15^N)	1024 (^1^H), 256 (^15^N)
^1^H-^13^C-HSQC	900	1.0	512	4.7 ppm (^1^H), 86.75 ppm (^13^C), 143 ppm (^15^N)	8.2 ppm (^1^H), 24 ppm (^13^C)	68 ms (^1^H), 47 ms (^13^C)	1024 (^1^H), 512 (^13^C)

Correlation between the imino proton resonance and the imino nitrogen was achieved through the ^1^J_N,H_ coupling in ^1^H,^15^N-BEST-TROSY experiments, where the transfer delay was set to 5.4 ms. These experiments were recorded on a 700 MHz NMR spectrometer from 10 to 45 °C (Farjon et al. 2009; Favier and Brutscher 2011; Solyom et al. 2013).

Detection of correlations between fast exchanging imino and amino groups was achieved in a ^1^H,^15^N-CPMG-NOESY experiment, conducted with a mixing time of 130 ms (Mueller et al. 1995).

Determination of base pairing schemes in the RNA was achieved with the help of a 2D-^1^H,^15^N-BEST-TROSY-HNN-COSY experiment, that reports on the hydrogen bond ^2h^J_N,N_-based correlation of nitrogen atoms acting in the hydrogen bond as donor and acceptor (Dingley and Grzesiek 1998; Schulte-Herbrüggen and Sørensen 2000; Lescop et al. 2010). It was acquired with a 30 ms ^15^N-^15^N transfer delay.

Furthermore, we performed a long range ^1^H,^15^ N-sfHMQC to correlate adenine N1/N3 nitrogens with H2-protons (transfer delay 20 ms).

CN-correlations in amino groups were achieved with a ^13^C-^15^N-HSQC (Schnieders et al. 2019).

To correlate amino protons to their respective nitrogen we performed ^1^H,^15^N-HSQC experiments with a relaxation and exchange optimized transfer delay of 4.6 ms (Mori et al. 1995).

Correlation within aromatic CH groups was achieved with an ^1^H-^13^C-HSQC experiment (INEPT transfer 2.7 ms) with off-resonant decoupling (Bodenhausen and Ruben 1980).

### Extent of assignment and data deposition

**Fig. 1:**
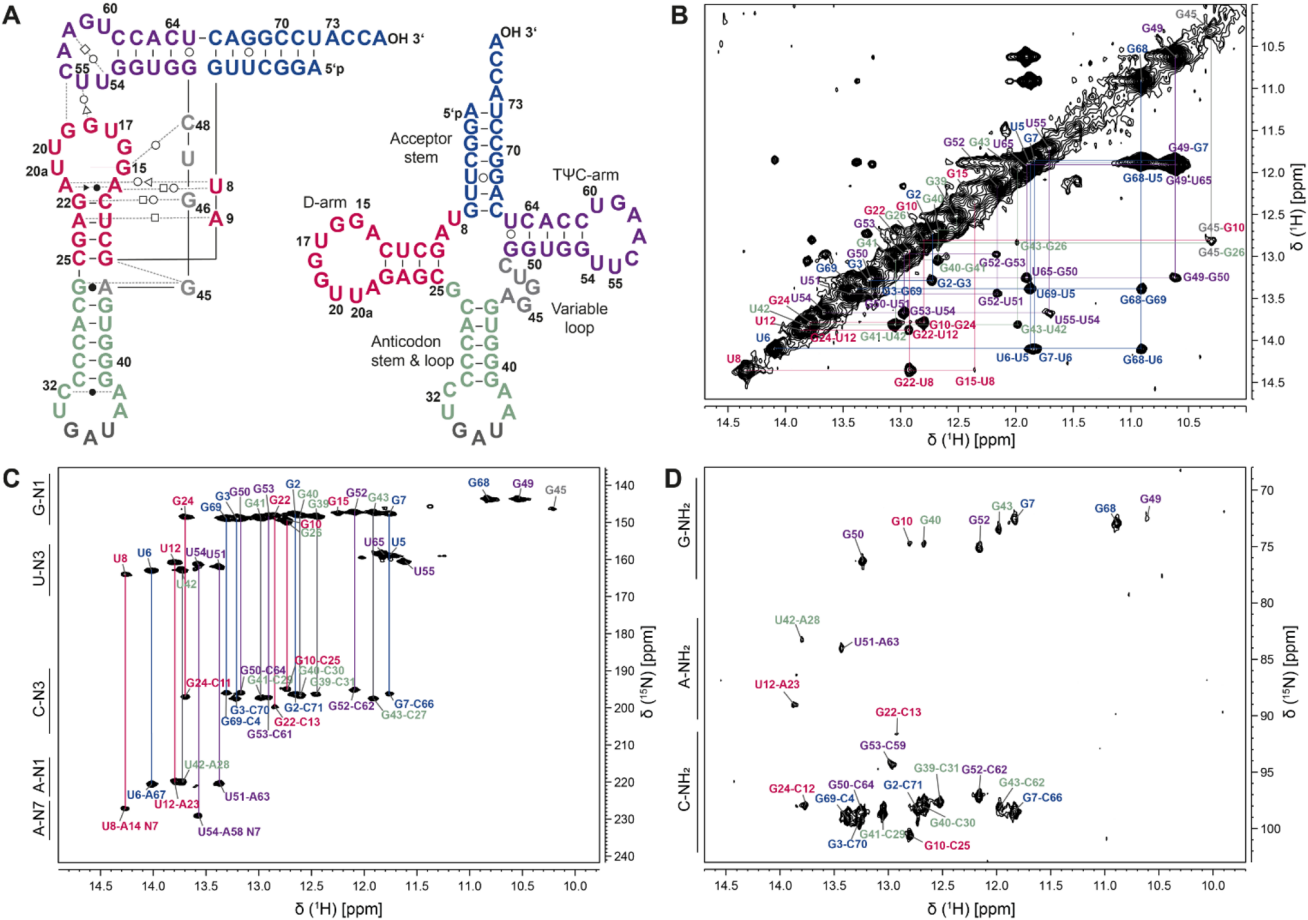
Assignment of tRNA^Ile^ from *E. coli*. **A** Secondary structure of tRNA^Ile^ shown in two representations. The acceptor stem is shown in blue, the D-arm in magenta, the anticodon stem and loop in green, the variable loop in grey and the TΨC-arm in purple. The three symbols are defined as follows: circle = Watson–Crick, square = Hoogsteen and triangle = sugar edge and are either in cis (filled) or trans conformation (open) (Leontis and Westhof 2001). Annotations in the spectra are color coded according to the secondary structure. **B** 2D ^1^H-^1^H-NOESY spectrum of tRNA^Ile^ shown for the assignment of the imino resonances. **C** ^1^H-^15^N-BEST-TROSY-HNN-COSY experiment of tRNA^Ile^ for the correlation of G-N1 to C-N3 and U-N3 to A-N1 and A-N7. **D** ^1^H-^15^N-CPMG-NOESY-HSQC spectrum to correlate NH_2_ groups

Before assigning the 77 nt *E. coli* tRNA^Ile^ (Fig. 1A), we acquired 2D-^1^H,^15^N-BEST-TROSY spectra at different temperatures ranging from 10 to 45 °C to elucidate at which temperature most of the exchangeable protons give rise to sharp and well dispersed signals. Furthermore, this temperature series allowed to deduce which base pairs are less stable and might stem from e. g. the closing base pairs of helices. Thereby, we found that at a temperature of 25 °C the spectra exhibited the most suitable signature of resonances to conduct assignment experiments. In total 29 well-resolved and intense imino resonances of guanosine and uridine nucleotides are visible in the spectra. All these resonances could be assigned unambiguously. Additionally, we observe four signals with a much-reduced intensity (1:10) which we attribute to a low populated second conformational state. With respect to the predicted secondary structure (Silvian et al. 1999), we could detect and assign all expected resonances of imino groups for base-paired nucleotides except for the A1:U72. The signal for this terminal base pair could be rendered beyond detectability due to dynamic motions in the ACCA-3’ end of the tRNA. Except for G19 and G46, we could detect imino proton resonances of all G and U nucleotides involved in tertiary interactions. The ^1^H-^15^N-BEST-TROSY-HNN-COSY experiment correlated G-N1 to C-N3 and U-N3 to A-N1 and A-N7. In addition, we could assign all detectable NH_2_ groups of paired Gs, As, and Cs through the application of an ^1^H-^15^N-CPMG-NOESY-HSQC (except for A67) (Fig. 1D). The protons of NH_2_ groups were further assigned by ^1^H-^15^N-HSQC selective for resonances stemming from the nucleobase’s amino groups. The use of ^1^H-^15^N-sfHMQCs allowed the correlation of adenine N1/N3 nitrogens to the H2-protons. Further, nitrogen to carbon correlations for amino groups were achieved with a ^13^C-^15^ N-HSQC, enabling the assignment of the C4 (for adenine and cytosine) and C2 (for guanosine) resonances. Correlation within aromatic CH groups in adenosines (C8H8 and C2H2) was achieved with a ^1^H-^13^C HSQC experiment.

The two G-U wobble base pairs served as starting point for the resonance assignment in the 2D ^1^H-^1^H-NOESY experiments at 10 °C and 25 °C (Fig. 1B) based on their strong intra-base pair NOEs. The imino protons of the U5:G68 base pair in the acceptor stem were assigned due to sequential NOEs to the imino proton of U6 in the U6:A67 base pair and the imino proton of G68 from the C4:G68 base pair. With this starting point the remainder of the imino protons from the other Watson–Crick base pairs in the acceptor stem were unambiguously assigned based on sequential NOEs apart from U72 residing within the terminal A1:U72 base pair. For U72 no imino proton was observable most likely due to fraying at the helix end. The imino protons of the G49:U65 wobble base pair at the apex of the TΨC-arm show sequential NOEs to the imino proton of G50. With this starting point, the base paired region of the TΨC-arm was unambiguously assigned (from G49-C65 to G53-C61). Additionally, the imino groups of nucleotides U54 and U55 could be assigned. These two residues are essential for the formation of the tertiary interactions within the TΨC-loop and with residues from the D-loop. U54 forms a reverse-Hoogsteen base pair with A58 (Zagryadskaya et al. 2003) highlighted by a cross peak in the HNN-COSY spectrum between the imino proton H3 and the nitrogen N7 acting as hydrogen bond acceptor nitrogen (229.4 ppm, ^15^N). The hydrogen bond acceptor of U55’s imino proton is the phosphate oxygen of A58 emphasized by the lack of a cross peak in the HNN experiment and a cross peak in a long range ^1^H-^31^P-HMQC experiment (Duchardt-Ferner et al. 2011; Duchardt-Ferner and Wöhnert 2017) to a phosphate resonance. However, the peak of the imino resonance is undetectable at 45 °C in the BEST-TROSY experiment. Both, the resonances of U55 and U54 are part of the walk of NOESY connectivities in the TΨC-arm of the tRNA.

Through the stacking of the acceptor stem and the TΨC-arm (Fig. 1A), nucleotides G49 and G7 come into close proximity. This is reported by a cross peak between their two imino proton resonances. The resonance of G7 is then also the starting point for a sequential walk of NOESY cross peaks through the acceptor stem that includes base pairs G7-C66 to G2-C72. This also corroborates the before mentioned assignment of the wobble base pair G68-U5.

The imino proton resonances stemming from the D-arm were assigned starting from G22 (G22, U12, G24 and G10). The imino proton of G10 showed an additional NOE contact to G45 in the variable loop. G45 has an NOE contact to G26 that is observable as a distinct and resolved peak in the NOESY spectrum at 10 °C (Westhof and Leontis 2021)**.**

The resonance of U8 is typically the most downfield shifted peak and was assigned at 14.27 ppm (^1^H) and 164.24 ppm (^15^N). Furthermore, its imino group is hydrogen bonded to N7 of A14, which is confirmed in the HNN-COSY spectrum (Fig. 1C). The close spatial proximity of U8 to G22 and G15 induces NOE cross peaks between their imino proton resonances, exhibiting a strong NOE between U8 and G22 and a weaker NOE between U8 and G15. The remaining G-U-G-G-G NOESY walk was assigned to the anticodon-stem-loop (ACSL) consisting of the Watson–Crick base pairs G43:C27 to G39:C31 (Fig. 1A).

In summary, for the imino proton resonances, we assigned 19 out of 22 base-paired G residues (86%) and 9 out of 10 base-paired U residues (90%). The corresponding hydrogen bond acceptor nuclei C-N3, A-N1 and A-N7 were all assigned by the ^1^H-^15^N-BEST-TROSY-HNN-COSY experiment. In addition, 25 amino groups were assigned (G-N2, C-N4 and A-N6, 42% in total). We assigned 28% of G-C2 and 74% of C-H41 and C-H42. Furthermore, for 4 out of 5 AU base pairs present in tRNA^Ile^, we assigned the corresponding A-N3, A-C2 and A-H2 resonances.

**Fig. 2:**
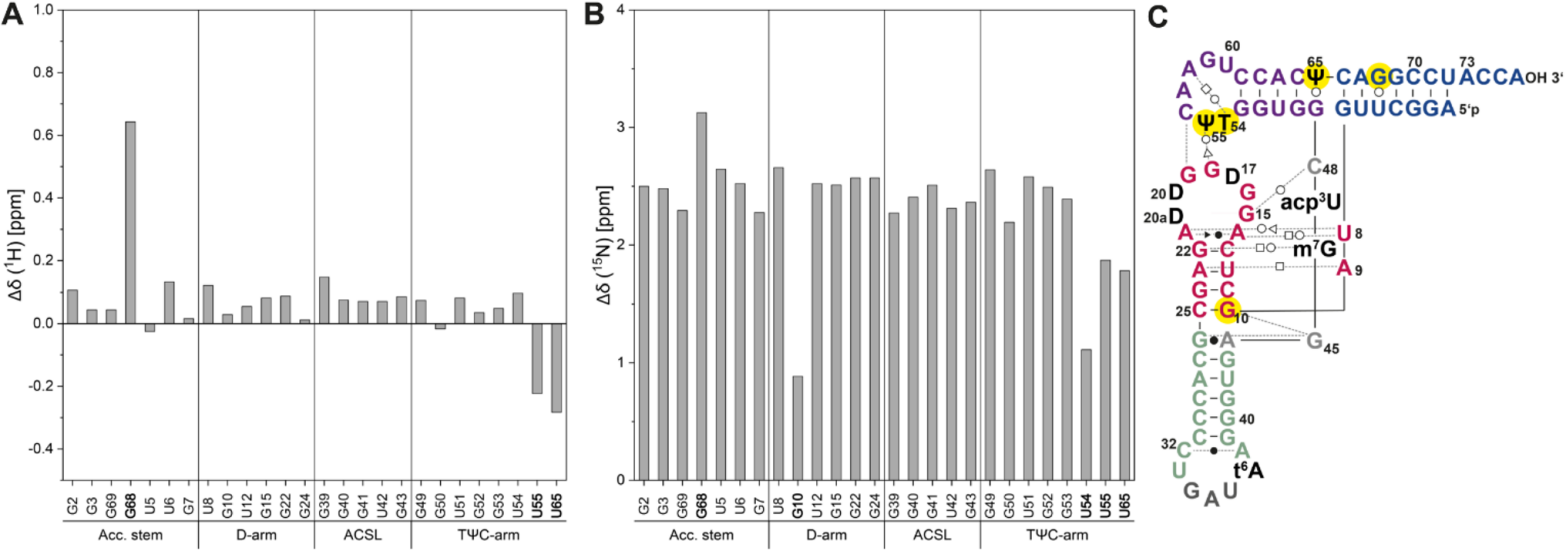
Chemical shift difference between the imino resonances of the non-modified and modified tRNA^Ile^ from *E. coli* construct was calculated as absolute values of the difference between corresponding imino signals for **A** ^1^H and ^15^N. Imino resonances of modified tRNA^Ile^ were taken from (Niimi et al. 1993) where the modified tRNA^Ile^ was measured in H_2_O, pH 6.7, 100 mM NaCl, 10 mM MgCl_2_ and 5% D_2_O and at 37 °C. Here, non-modified tRNA^Ile^ was investigated in 25 mM KPi, pH 6.2, 200 mM KCl, 5 mM MgCl_2_, and 5% D_2_O. **C** The modified tRNA^Ile^ is shown in the L-shaped secondary structure. The modified residues are shown in black. The residues with the highest chemical shift difference are highlighted in yellow

Based on these assignments we could determine the chemical shift differences between modified tRNA^Ile^ (Niimi et al. 1993) and non-modified tRNA^Ile^ as a first step towards the analysis of the effect of nucleotide modifications on tRNA’s structure and dynamics. The chemical shift differences are shown in Fig. 2. The most prominent differences in chemical shifts are observed for residues close to the helix junctions (e.g. G10) and for the nucleotides that establish the tertiary interaction between the D-arm and the TΨC loop, where also the nucleobase modifications T54 and Ψ55 occur. In addition, residue 65 shows a large chemical shift difference, as U65 is modified to Ψ65 in the modified tRNA^Ile^. It is noteworthy to mention that the compared spectra were referenced differently, resulting in an absolute chemical shift difference of ~ 2.3 ppm in the ^15^N dimension. Although there are small differences in the buffer composition and for the temperature in which both tRNAs were measured, the structure of the tRNA is not perturbed and therefore chemical shifts are comparable.

The chemical shifts for tRNA^Ile^ have been deposited in the Biological Magnetic Resonance Bank (http://www.bmrb.wisc.edu) with the Accession Number 51164.

## Data Availability

Chemical shift data is available at the Biological Magnetic Resonance Bank (http://www.bmrb.wisc.edu).
